# Incorporating Electrostatic Coupling Effects into Multispecies Solute Transport Simulations with MODFLOW

**DOI:** 10.1111/gwat.70033

**Published:** 2025-11-28

**Authors:** Rodrigo Pérez‐Illanes, Christian D. Langevin, Muhammad Muniruzzaman, Massimo Rolle

**Affiliations:** ^1^ Aquatic Geochemistry Group, Institute of Applied Geosciences Technical University of Darmstadt Schnittspahnstraße 9 64287 Darmstadt Germany; ^2^ S.S. Papadopulos & Assoc., Inc. 12 Dove Ln Saint Paul MN 55127; ^3^ Institute of Geosciences University of Bonn Kirschallee 1‐3 53115 Bonn Germany; ^4^ Water Management Unit Geological Survey of Finland Vuorimiehentie 5 Espoo 02151 Finland

## Abstract

Solute transport simulators aiming to accurately describe the transport of charged chemical species in porous media need to account for electrostatic coupling effects. Each ion in pore water possesses a specific electric charge and molecular diffusion coefficient, properties that determine their mobility and the overall charge balance of aqueous solutions. Depending on the charge, concentration and aqueous diffusion coefficient, the displacement of an ion in solution influences, and is in turn influenced by, other ions in solution by means of electrostatic interactions. This phenomenon has been studied with experiments and numerical simulations in diffusion‐dominated regimes, as well as in advection‐dominated flow‐through systems, showing that electrostatic coupling effects play a relevant role in the spatiotemporal prediction of ion concentrations. However, there is limited availability of solute transport codes incorporating electrostatic coupling, limiting applications of multispecies ionic transport at different scales. This article elaborates on the topic of electrostatic coupling and presents a methodology for incorporating the effect into multispecies solute transport simulations with MODFLOW. The integration is achieved through the Application Programming Interface of the program (MODFLOW‐API). This interface enables the access to concentrations and dispersion coefficients of all species during the simulation, which are necessary to calculate a dispersive correction that effectively incorporates electrostatic coupling into the model. Numerical results demonstrate the effectiveness of the coupling strategy, benchmarking the implementation with previously validated numerical simulators and with experimental data.

## Introduction

The macroscopic description of solute transport through porous media is fundamentally influenced by processes occurring at microscopic scales. Differences in molecular diffusion coefficients have been shown to explain notable macroscopic differences in the extent of transverse mixing (e.g., Chiogna et al. 2010; Pérez‐Illanes et al. 2024), as well as in the breakthrough curves of different chemical species (e.g., Rolle and Kitanidis 2014; Muniruzzaman and Rolle 2017). The dispersive transport of ionic (charged) species can be further influenced by electrostatic interactions with other ionic species in solution (e.g., Carey et al. 1995; Boudreau et al. 2004; Liu et al. 2011; Rolle et al. 2013), or influenced by surface charges associated with the material composition of the porous matrix (e.g., Appelo et al. 2008; Steefel and Tournassat 2020). These processes are studied in the context of multispecies ionic solute transport, and have been shown to be of relevance for a variety of reactive transport applications in porous media. Accounting for electrostatic interactions is relevant for modeling transport through clays, where ion concentrations also interact with the surface charge of the porous matrix (e.g., Appelo and Wersin 2007; Appelo et al. 2008; Soler et al. 2019; Steefel and Tournassat 2020; Muniruzzaman and Rolle 2021), in electrokinetic simulations where transport is governed by the application of an external electric field (e.g., Sprocati et al. 2019; Sprocati et al. 2020; López‐Vizcaíno et al. 2022), in studies discussing mineral weathering in hydrothermal systems (e.g., Giambalvo et al. 2002), and pollutant diffusion and back‐diffusion through porous media (e.g., Liu et al. 2011; Muniruzzaman and Rolle 2019).

There have been efforts to include electrostatic interactions in the solute transport formulation of some computer codes (Steefel et al. 2014). For example, one‐dimensional multidiffusion simulations accounting for electrostatic interactions are possible with the geochemical software PHREEQC (Parkhurst and Appelo 2013; Rolle et al. 2013), electrostatic interactions with the surface charge of clay materials are included in CrunchClay (Steefel and Tournassat 2020), electrostatic coupling was discussed for the code MIN3P (Rasouli et al. 2015), Coulombic interactions were accounted for in applications coupling the PhreeqcRM reaction engine (Parkhurst and Wissmeier 2015) with COMSOL Multiphysics® (e.g., Rolle et al. 2018; Sprocati et al. 2019), more recently a Nernst‐Planck formulation of transport was discussed for the reactive transport code PFLOTRAN^NP^ (Trinchero et al. 2022), and electrostatic interactions coupled with density effects were also considered in simulations with RetroPy (Huang et al. 2023). This work describes an approach for incorporating electrostatic coupling effects into multispecies solute transport simulations with MODFLOW (Langevin et al. 2017; Langevin et al. 2022), a well‐known open‐source hydrogeologic simulator. The program incorporates a large variety of flow and transport processes relevant for the study of regional‐scale aquifers (Langevin et al. 2023), and therefore, adding this new capability will extend its applicability to a wider range of problems where electrostatic coupling effects are known to be of relevance (e.g., Appelo and Wersin 2007; Rasouli et al. 2015; Charlet et al. 2017; Tournassat and Steefel 2019; Muniruzzaman and Rolle 2021). To our knowledge, incorporating the effects of electrostatic coupling into MODFLOW has not yet been addressed in the literature.

This work is framed in the same context of recent contributions implementing innovative modeling approaches for computer programs utilized in studies of subsurface water quality (e.g., Wu et al. 2015; Atteia et al. 2023; Pérez‐Illanes and Fernàndez‐Garcia 2024). Along this line, the main goal of this study is to develop a methodology that incorporates electrostatic coupling effects into multispecies solute transport simulations performed with the transport module of MODFLOW (also known as GWT). Electrostatic interactions are incorporated into a simulation by taking advantage of the functionalities provided by the recently released Application Programming Interface (API, MODFLOW‐API; Hughes et al. 2022). The API allows an external program or script to access and modify internal MODFLOW variables while the program is running a simulation. This allows users of the MODFLOW‐API to intercept the temporal evolution of simulations, which among other applications, can be used to incorporate additional physical processes into the native MODFLOW solver. This approach has been shown to be effective in making use of the solute transport capabilities of the program for reactive transport applications with a nonlinear dispersion model (Pérez‐Illanes et al. 2024). Furthermore, the flexibility provided by the API enables fast prototyping of new applications making use of the MODFLOW solver (as in White et al. 2023; Larsen et al. 2024), and hence, it is the platform selected in this work to evaluate the incorporation of electrostatic coupling effects into multispecies solute transport simulations with the program.

The proposed MODFLOW‐API workflow incorporating electrostatic coupling and its implementation is described in the following. Its performance is later evaluated via comparison with benchmark numerical simulations accounting for electrostatic coupling that were previously validated in the literature with experimental data.

## Methods

### Electrostatic Interactions

Electrostatic coupling effects are expected to occur during the transport of ionic (charged) chemical species, and are influenced by the magnitude of ion concentrations and two other distinctive properties of ions in aqueous solution. First, each ion diffuses with its own specific molecular diffusion coefficient, determining its rate of mixing. Secondly, each ion has its own specific electric charge. This means that during transport ion concentrations will influence local charge balance, impacting in turn the transport of other ionic species in solution by means of electrostatic interactions. In the absence of an external electric field, the condition of electroneutrality (neutral charge balance) must be satisfied throughout the pore water

(1) $$\sum_{i=1}^{N_{c}}z_{i}c_{i}=0,$$

where $z_{i}$ and $c_{i}$ are the electric charge and concentration of a species i respectively, and $N_{c}$ is the total number of species in solution. The electroneutrality condition has been shown to be equivalent to the condition of zero electric flux (Boudreau et al. 2004), which is written as

(2) $$\sum_{i=1}^{N_{c}}z_{i}J_{i}=0,$$

where $J_{i}$ is the diffusive flux of a species i. For ionic species, the diffusive flux $J_{i}$ is not only influenced by chemical concentration gradients, but also by electrostatic interactions with other ions. Assuming dilute solutions and unit chemical activities for simplicity, the diffusive flux of a charged species is written as

(3) $$J_{i}=-{𝒟}_{i}\nabla c_{i}-{𝒟}_{i}\frac{z_{i}F}{\mathrm{RT}}c_{i}\nabla \Phi ,$$

where ${𝒟}_{i}$ is the aqueous molecular diffusion coefficient of species i, F is the Faraday constant, R the ideal gas constant, T the temperature and ∇Φ the gradient of electrostatic potential. The form of diffusive flux in Equation (3) is usually known as the Nernst‐Planck formulation (e.g., Steefel et al. 2014; Rolle et al. 2018; Tournassat et al. 2020; Trinchero et al. 2022). The first component of the flux in Equation (3) is due to chemical concentration gradients, while the second component is controlled by gradients in the electrostatic potential ∇Φ, taken to be the same for all species in solution. By imposing the zero electric flux condition (Equation 2) using the diffusive flux expression previously discussed (Equation 3), an equivalence for the gradient of electrostatic potential in terms of the concentrations and diffusion coefficients of all the species in solution can be found as

(4) $$\nabla \Phi =\frac{\sum_{i=1}^{N_{c}}z_{i}{𝒟}_{i}\nabla c_{i}}{\sum_{j=1}^{N_{c}}\left(z_{j}^{2}{𝒟}_{j}c_{j}F\right)/\mathrm{RT}}.$$

Substituting Equation (4) back into expression (3), the flux is effectively rewritten as a function of the concentrations (gradients), and diffusion coefficients of all species. In the context of advection‐dispersion through porous media, the transport equation arising from a Nernst‐Planck formulation of fluxes is given as (e.g., Rolle et al. 2018)

(5) $$\frac{\partial \left(\phi c_{i}\right)}{\partial t}+\nabla \cdot \left(qc_{i}\right)=\nabla \cdot \left(\phi \sum_{j=1}^{N_{c}}D_{\mathrm{ij}}\nabla c_{j}\right),$$

where ϕ is the medium porosity, q is the specific discharge vector, and $D_{\mathrm{ij}}$ is a coupled dispersion tensor. In streamline‐oriented formulations, where the system is oriented along the principal directions, the longitudinal and transverse coupled dispersion coefficients are given respectively by (Rolle et al. 2013; Muniruzzaman et al. 2014)

(6) $$D_{\mathrm{ij}}^{L}=\delta_{\mathrm{ij}}D_{i}^{L}-\frac{z_{i}z_{j}D_{i}^{L}D_{j}^{L}c_{i}}{\sum_{k=1}^{N_{c}}z_{k}^{2}D_{k}^{L}c_{k}},$$

(7) $$D_{\mathrm{ij}}^{T}=\delta_{\mathrm{ij}}D_{i}^{T}-\frac{z_{i}z_{j}D_{i}^{T}D_{j}^{T}c_{i}}{\sum_{k=1}^{N_{c}}z_{k}^{2}D_{k}^{T}c_{k}},$$

where $\delta_{\mathrm{ij}}$ is the Kronecker delta, and $D_{i}^{L}$ and $D_{i}^{T}$ are respectively the longitudinal and transverse dispersion coefficients for species i in its liberated state (i.e., electrostatically uncoupled). There are two important aspects to remark here. First, that the dispersive part of the transport equation for species i (right‐hand‐side in Equation 5) is now written in terms of the concentration gradients of all species in solution. This means that, by default, there is a tight coupling between all the species in order to solve the transport equation for one of them. Secondly, that the coupled dispersion coefficients (Equations 6 and 7) entail the dispersion of the species in its liberated state plus an additional electrostatic coupling term which is calculated as a function of all the electric charges, all dispersion coefficients, and all charged species' concentrations in solution, meaning that Equation (5) is effectively a nonlinear transport equation. In practice, codes aiming to implement this kind of coupling need to keep track of all the concentrations at all time steps, which will determine as well the magnitude of the coupled dispersion coefficient at that instant. These two characteristics motivate the application of a *lagged‐one‐time‐step* approach to solve the transport equation of multiple interacting ionic chemical species. In general, this lagged approach is a reasonable (and straightforward) alternative for solving nonlinear diffusion problems (Özişik et al. 2017), and its accuracy could be easily evaluated by systematically reducing the simulation time step. Furthermore, this same approach has been previously shown to be effective in making use of the MODFLOW solute transport solver for reactive transport applications with a nonlinear dispersion model (Pérez‐Illanes et al. 2024).

### Dispersion Coefficients

The molecular diffusion coefficient is known to be specific to each chemical species and can be determined with empirical correlations (Wilke and Chang 1955; Worch 1993) or experimentally (Jin et al. 2014; Urík et al. 2020). Experimental and numerical studies in porous media (both at the pore and macroscopic scales) have shown substantial differences in the quantitative metrics of conservative and reactive solute transport for different chemical species (e.g., Rolle et al. 2012; Hochstetler et al. 2013; Wienkenjohann et al. 2023), illustrating that besides the influence of the grains distribution and flow patterns, hydrodynamic dispersion is also impacted by the differences in molecular diffusion coefficients. In this study, we use a local parameterization of the longitudinal and transverse dispersion coefficients developed and validated in high‐resolution flow‐through experiments in granular porous media (Guedes de Carvalho and Delgado 2005; Chiogna et al. 2010; Ye et al. 2015; Kurotori et al. 2019)

(8) $$D_{i}^{L}={𝒟}_{i}^{p}+\frac{1}{2}\mathrm{dv},$$

(9) $$D_{i}^{T}={𝒟}_{i}^{p}+{𝒟}_{i}{\left(\frac{{\mathrm{Pe}}_{i}^{2}}{{\mathrm{Pe}}_{i}+2+4\delta^{2}}\right)}^{\beta_{T}},$$

where ${𝒟}_{i}^{p}$ is the pore diffusion coefficient (${𝒟}_{i}$ corrected for tortuosity) commonly approximated by the simple relationship ${𝒟}_{i}^{p}\approx \phi {𝒟}_{i}$, v is the flow velocity, d a characteristic grain size of the porous medium, ${\mathrm{Pe}}_{i}=\mathrm{vd}/{𝒟}_{i}$ is the Péclet number for a species i, δ is a parameter describing the ratio between the length of a pore channel and its hydraulic radius (Bear 1979), and $\beta_{T}$ is a nonlinear exponent to model the extent of incomplete mixing inside a pore channel (Chiogna et al. 2010; Ye et al. 2015). In the case of the transverse direction, the advantage of a dispersion model like Equation (9) is that it explicitly retains the influence of the different molecular diffusion coefficients, given the same groundwater flow conditions and porous medium. The dispersion coefficients of Equations (8) and (9) can be given to the transport solver in a straightforward manner by calculating the equivalent dispersivities for a species i (Basilio Hazas et al. 2023; Pérez‐Illanes et al. 2024; Ye et al. 2025). This approach is implemented here for ionic species and the following equivalent dispersivities are provided to the dispersion package of the MODFLOW transport solver

(10) $$\alpha_{L,i}^{*}=\frac{1}{2}d,$$

(11) $$\alpha_{T,i}^{*}=\frac{d}{{\mathrm{Pe}}_{i}}{\left(\frac{{\mathrm{Pe}}_{i}^{2}}{{\mathrm{Pe}}_{i}+2+4\delta^{2}}\right)}^{\beta_{T}},$$

where $\alpha_{L,i}^{*}$ and $\alpha_{T,i}^{*}$ are the longitudinal and transverse equivalent dispersivities for species i, respectively. Taking the transverse component for illustration purposes, MODFLOW will then internally evaluate hydrodynamic dispersion following an expression of the form $D_{i}^{T}={𝒟}_{i}^{p}+\alpha_{T,i}^{*}v$, effectively incorporating the model of Equation (9) into the MODFLOW solver. In simulations where the groundwater flow velocity is also expected to change in time, one could make use of the API to extract the real‐time flow velocities and update the equivalent dispersivities to incorporate these transient changes into the parameters of the transport simulation.

**Figure 1:**
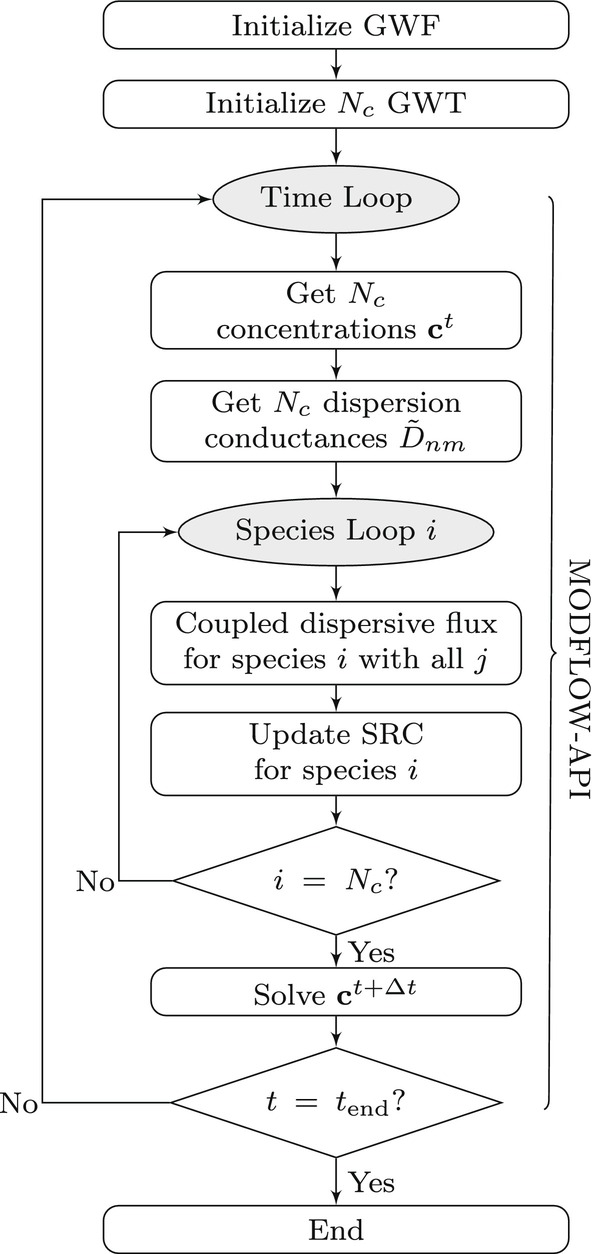
Workflow diagram for implementing electrostatic coupling effects into a multispecies GWT simulation using the MODFLOW‐API with a *lagged‐one‐time‐step* approach.

**Table 1:** MODFLOW Memory Addresses Queried (Get) or Modified (Set) with the MODFLOW‐API for the Electrostatic Coupling of Dispersion

Memory Address	Interpretation	API
{GWF}/CON/NODES	Number of cells	Get
{GWF}/CON/IA	Connectivity indexes	Get
{GWF}/CON/JA	Connected cells	Get
{GWF}/CON/JAS	Connected cells	Get
{GWTi}/X	Concentration	Get
{GWTi}/DSP/DISPCOEF	Disp. conduc.	Get
{GWTi}/CONCENTRATION/BOUND	Source term	Set

Note: Curly brackets indicate replacement by the name of the corresponding groundwater flow model (GWF), and the name of the transport model for species i (GWTi).

### Implementation

Electrostatic coupling effects are incorporated into MODFLOW‐based multispecies transport simulations via the use of the MODFLOW‐API, following the proposed workflow shown in Figure 1. For reference, the memory addresses of the internal MODFLOW variables employed to achieve the implementation are given in Table 1. The method relies on a *lagged‐one‐time‐step* approach, meaning that the additional dispersive fluxes representing the electrostatic coupling of one species with all the others are evaluated from the $N_{c}$ concentrations and dispersion coefficients of the previous time step. The contribution of these fluxes to the mass balance of a species is calculated outside of MODFLOW's source code, and then given as a source term to the corresponding solute transport model via the Mass Source Loading package (SRC; Langevin et al. 2022). In general, this lagged approach can be implemented in a straightforward manner with the API, and it avoids rewriting the matrix of coefficients for a given species, or otherwise creating many interaction terms (called exchanges in MODFLOW terminology) between all species. Though it would be possible within the MODFLOW framework to simultaneously solve all chemical species in a single system of equations for each time step, the large number of unknowns could be cost‐prohibitive for typical applications.

The simulation workflow begins by first initializing a MODFLOW groundwater flow model (GWF), which in the context of this work is always taken to be at steady‐state (though the extension to transient flow conditions is straightforward). Then, $N_{c}$ different MODFLOW solute transport models (GWT) are configured (one for each chemical species). Each solute transport model has its own specific transport coefficients, based on the evaluation of species‐specific dispersion parameters (Equations (8), (9), (10), (11)). Each GWT model is configured to have a SRC package initially acting as a placeholder that will then be updated with the contribution to mass balance of the additional Nernst‐Planck‐based dispersive fluxes incorporating electrostatic coupling.

As an initial approach, the evaluation of the electrostatic coupling terms is performed following the *Simplified Formulation* of dispersion implemented in MODFLOW. This approach is recommended for transport simulations in which the flow velocity is generally aligned with the principal directions of the grid (for a discussion, refer to Langevin et al. 2022). The advantage of this simplification is that it allows to implement and evaluate the performance of the API workflow for incorporating electrostatic coupling (Figure 1) in a relatively straightforward manner, at the cost of potentially restricting the applicability of the implementation to simple groundwater flow configurations. The effectiveness of the coupling strategy is assessed later in this paper via comparison of the MODFLOW‐based multispecies transport simulations following the API workflow (Figure 1) with known numerical results accounting for electrostatic coupling that were previously validated in the literature with experimental data. At this point it is important to note that we allow MODFLOW to calculate the traditional species‐specific dispersive flux (first term in the right‐hand‐side of Equations 6 and 7). The electrostatic coupling is represented with the API by evaluating the additional dispersive flux experienced by a species i due to the concentration gradients of all the other species j (second term in the right‐hand‐side of Equations 6 and 7), and by giving these fluxes to the solute transport solver as a source term.

For each species i and flow‐model cell n, the objective is to evaluate a source term of the form

(12) $${\mathrm{SRC}}_{i;n}=\sum_{j=1}^{N_{c}}\sum_{m\in \eta_{n}}F_{i,j;n,m},$$

where $\eta_{n}$ is the set of cell indices m connected to cell n, and $F_{i,j;n,m}$ is the coupled dispersion flux for species i due to the concentration gradient of species j between cells n and m, evaluated in the context of the *Simplified Formulation* of dispersion as (Langevin et al. 2022)

(13) $$F_{i,j;n,m}={\tilde{D}}_{i,j;n,m}\left(c_{j;m}-c_{j;n}\right),$$

where $c_{j;n}$ is the concentration of species j in cell n, and ${\tilde{D}}_{i,j;n,m}$ is the dispersive conductance incorporating electrostatic coupling, calculated as

(14) $${\left.{\tilde{D}}_{i,j;n,m}=-\frac{z_{i}z_{j}{\tilde{D}}_{i}{\tilde{D}}_{j}{\bar{c}}_{i}}{\sum_{k=1}^{N_{c}}z_{k}^{2}{\tilde{D}}_{k}{\bar{c}}_{k}}\right|}_{n,m}.$$

In this expression, the dispersive conductances for species i and j (${\tilde{D}}_{i}$ and ${\tilde{D}}_{j}$) were evaluated natively by MODFLOW at the interface connecting cells n and m, and can be obtained from the internal memory with the API (Table 1). These conductances already incorporate the harmonic average between the specific transport properties of the cells connected at the interface, following the calculations discussed in the documentation of the program (Langevin et al. 2022). The bar over concentrations in Equation (14) indicates that the value of the concentration at the interface is interpolated from the concentration in cells n and m (for a discussion refer to Tournassat et al. 2020).

During the development of the proposed API workflow to incorporate electrostatic coupling, it became evident that the internal cell‐connectivity arrays of MODFLOW (Table 1) were going to be needed in order to evaluate the additional dispersive effect (as seen in Equations 12 and 13). Although this calculation is performed outside of MODFLOW (in the external script orchestrating the API workflow), its implementation follows closely the Fortran source code of the program. One positive consequence of making use of the internal cell‐connectivity arrays in the implementation, is that the API workflow with electrostatic coupling is fully compatible with any flow model of any spatial dimensionality already handled by MODFLOW.

## Results and Discussion

**Figure 2:**
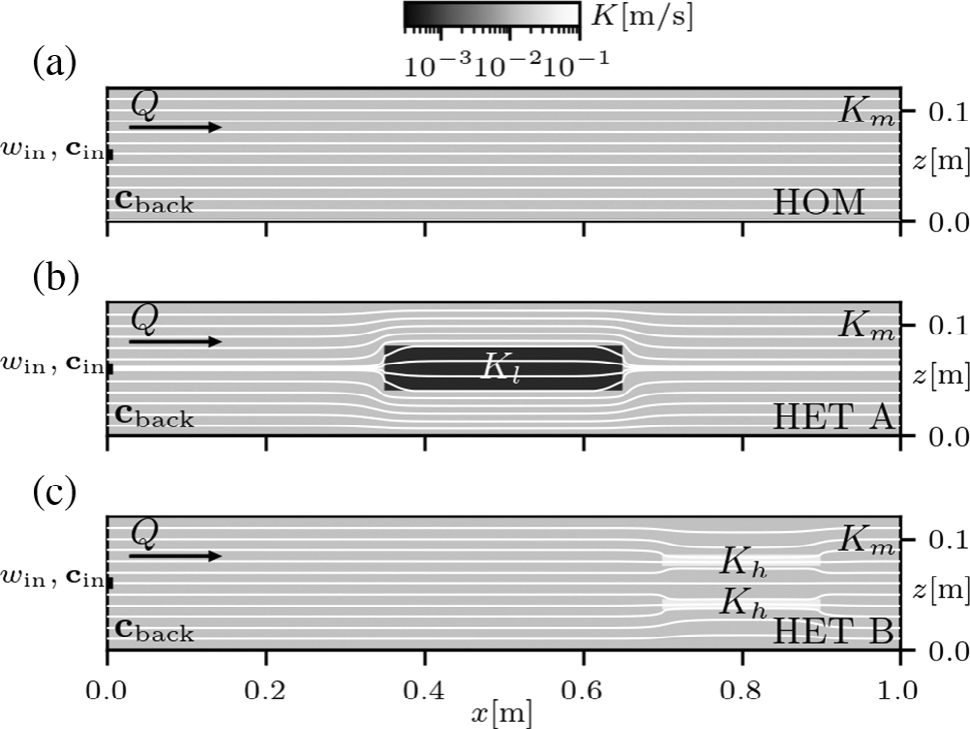
Diagram of experimental flow‐through systems employed in simulations. Panel (a) is a homogeneous medium with hydraulic conductivity $K_{m}$, panel (b) is a medium with a lower‐permeability inclusion $K_{l}$ (HET A), and panel (c) is a medium with two higher‐permeability inclusions $K_{h}$ (HET B). Horizontal lines represent advective streamlines calculated for each medium. Each medium is initially saturated with uniform background concentrations ($c_{\text{back}}$) and a tracer solution with concentrations $c_{\text{in}}$ is injected through a line source of width $w_{\text{in}}$.

This section presents results from simulations benchmarking the proposed MODFLOW‐based multispecies solute transport model incorporating electrostatic coupling, performed considering different boundary conditions and background electrolyte concentrations, in homogeneous and heterogeneous porous media (Figure 2). Table 2 summarizes the geometrical and physical properties of the considered setups, based on the configuration of lab‐scale experiments found in the literature. The results are compared to known simulations previously validated with analytical solutions, as well as high‐resolution experimental data from multidimensional flow‐through setups. All simulations were performed with the development version of MODFLOW@6.7.0.

**Table 2:** General Properties and Dimensions of the Flow‐Through Systems Employed in MODFLOW‐Based Numerical Simulations

Property		Value	Unit
Length	L	1.0	m
Height	H	0.12	m
Width	B	0.01	m
Porosity	ϕ	0.41	—
Hyd. Cond. med.	$K_{m}$	1.27 $\times 10^{-2}$	m/s
Hyd. Cond. low.	$K_{l}$	6.14 $\times 10^{-4}$	m/s
Hyd. Cond. high.	$K_{h}$	4.12 $\times 10^{-2}$	m/s
Grain diam. med.	$d_{m}$	1.13	mm
Grain diam. low.	$d_{l}$	0.25	mm
Grain diam. high.	$d_{h}$	2.03	mm
Line source inj.	$w_{\text{in}}$	{1,2}	cm
Cell size	Δx,Δz	0.1	cm

### Transport through Homogeneous Media

The first validation is performed considering a simple homogeneous porous medium (Figure 2a), with different scenarios of background electrolyte concentrations and continuous injection of tracer solutions. Two‐dimensional MODFLOW simulations are advanced until steady‐state transport conditions are achieved. The results are then compared with available one‐dimensional transient models with PHREEQC simulating lateral mixing at the outlet of the flow‐through system, which were previously validated in the literature with experimental data (Rolle et al. 2013). The numerical model simulates a two‐dimensional flow‐through system subjected to a homogeneous steady‐state flow condition. MODFLOW simulation of ionic transport accounts for electrostatic coupling, transporting as many solutes as ions in solution, each with different charge and specific diffusion coefficient. Strong electrolytes are considered as tracer solutions for the multispecies ionic transport simulation (e.g., NaCl or ${\text{MgCl}}_{2}$). The tracer injection is from a line source with a extent of $w_{\text{in}}=1$ cm located in the middle of the inlet boundary. Two conditions of background electrolyte concentration are simulated: (i) medium saturated with deionized water (e.g., Milli‐Q water), in which case it is expected that salt ions disperse together due to their electrostatic coupling, and (ii) a medium saturated with a strong background electrolyte solution (e.g., NaBr) at higher concentration than the injected salt tracer, allowing for the evaluation of differences in the transverse dispersion of ionic species. In the first case, tracer salt ions disperse together preserving a neutral charge balance and it is possible to calculate an apparent (unique) dispersion coefficient for the coupled ions (e.g., Cussler 2009). In the second condition, charge balance is mostly controlled by the background electrolyte solution, which is at a much higher concentration than the injected tracer. This means that the tracer salt ions do not need to be necessarily coupled to satisfy the electroneutrality condition (Equation 1); the background electrolyte behaves as a buffer and tracer salt ions can displace closer to their liberated (uncoupled) state.

**Figure 3:**
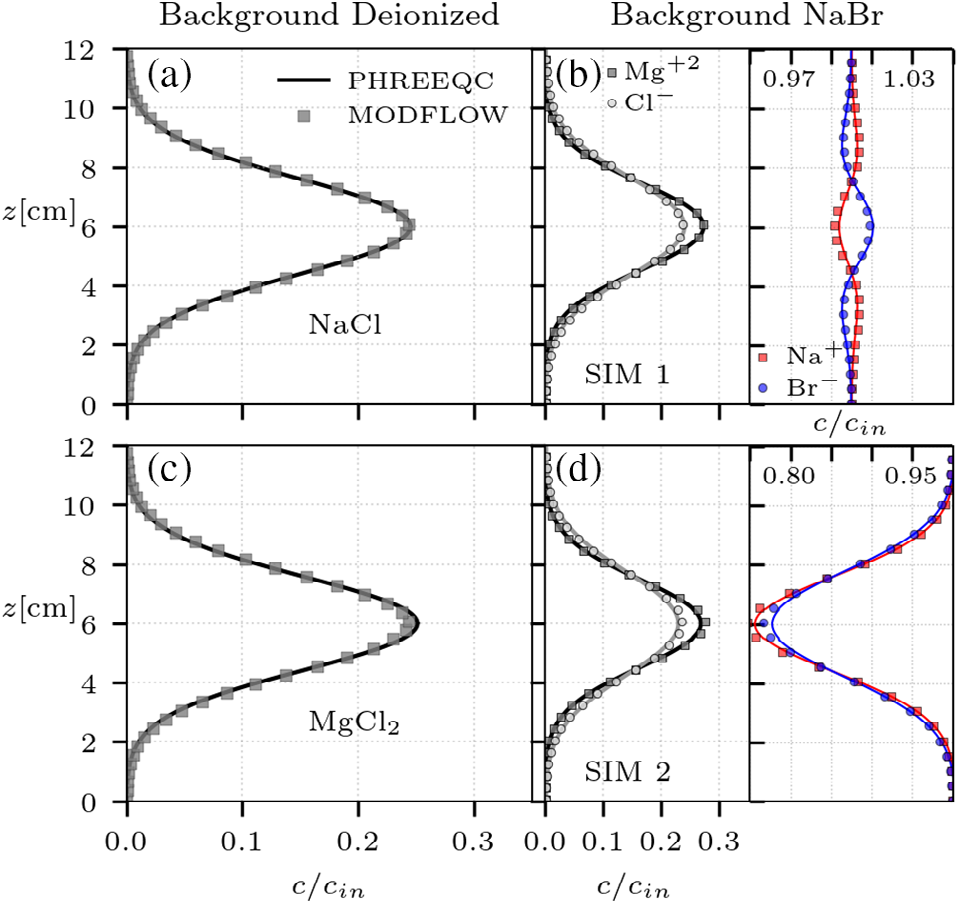
Comparison between the one‐dimensional transient PHREEQC simulations (solid lines) in homogeneous media and equivalent two‐dimensional models in MODFLOW (symbols). For simulations with background deionized water (a, c), panels display the transverse profile for only one ion, since the displacement of both ions is the same due to electrostatic coupling. For simulations with background NaBr solution (b, d) panels are subdivided for a specific range of normalized concentrations. Left subpanels show ion concentrations for the tracer salt (MgCl_2_) and right subpanels for the background electrolyte (NaBr). All concentrations are normalized by their respective initial value $c_{\text{in}}$.

Figure 3a and 3c presents the comparison between the one‐dimensional PHREEQC and two‐dimensional MODFLOW models simulating the continuous injection of a tracer salt solution into deionized pore water environment. Salts NaCl and MgCl_2_ are considered as tracers. The MODFLOW solute transport model accounts for $N_{c}=2$ solutes (one per ion of the tracer salt) and electrostatic interactions. Molecular diffusion coefficients and charges of each ion are given in Table 3. Transverse dispersion coefficients are calculated following expression (9) by giving to the respective MODFLOW solute transport model (GWT) an equivalent dispersivity (Equation 11) calculated with the species‐specific diffusion coefficient ${𝒟}_{i}$, and parameters $\beta_{T}=0.47$, δ=6.2, and d=1.25 mm (Rolle et al. 2013). Longitudinal dispersion is not playing a relevant role in this steady‐state transport problem. Flow velocity is uniform and horizontal with a value of v=1.5 m/d.

**Table 3:** Physical Properties of Ions in the Multispecies Ionic Transport Model

Species	Charge z	Diffusion 𝒟 ($m^{2}/s$)
Mg^+2^	+2	$0.63\times 10^{-9}$
Cl^−^	−1	$1.81\times 10^{-9}$
Na^+^	+1	$1.20\times 10^{-9}$
Br^−^	−1	$1.86\times 10^{-9}$

Note: Aqueous diffusion coefficients are representative of temperature T=20°C (Muniruzzaman and Rolle 2017).

The two‐dimensional MODFLOW‐based simulations are in good agreement with the reference one‐dimensional PHREEQC results, leading to the physically expected behavior of ions displacing at the same rate. This is seen for simulations of both the 1:1 electrolyte (NaCl, Figure 3a) and the 1:2 electrolyte (MgCl_2_; Figure 3c), verifying as well that the tracer salt ions, although characterized by different molalities and molecular diffusion coefficients, followed the same spatial distribution effectively representing electrostatic coupling. A more complex condition is simulated, now with the system saturated with a buffer electrolyte solution at a higher concentration than the injected tracer salt. NaBr is taken as background electrolyte, and two different scenarios of continuous injection are discussed: (i) the injection releases a mixed solution composed of both ${\text{MgCl}}_{2}$ and NaBr (labeled as SIM 1), and (ii) the injection is of pure ${\text{MgCl}}_{2}$ solution (labeled as SIM 2). In both these cases the background electrolyte concentration is about four times higher than the injected tracer concentration. The MODFLOW‐based model accounts for $N_{c}=4$ different species following the parameters shown in Table 3. The comparison between the PHREEQC reference (solid lines in Figure 3) and MODFLOW‐based results (symbols in Figure 3) demonstrates that electrostatic interactions were accurately incorporated into the model and that the coupling strategy is capable of handling complex conditions like those found while considering a strong background electrolyte solution. Furthermore, these simulations show that the MODFLOW‐based model is able to incorporate electrostatic coupling effects across different concentrations, as evidenced for example by analyzing the influence of the tracer on the distribution of the background electrolyte concentrations (Figure 3b and 3d). The model is capable of reproducing small scale effects in background concentrations, while also properly representing the transport of the injected tracer ions in all the simulated scenarios.

**Figure 4:**
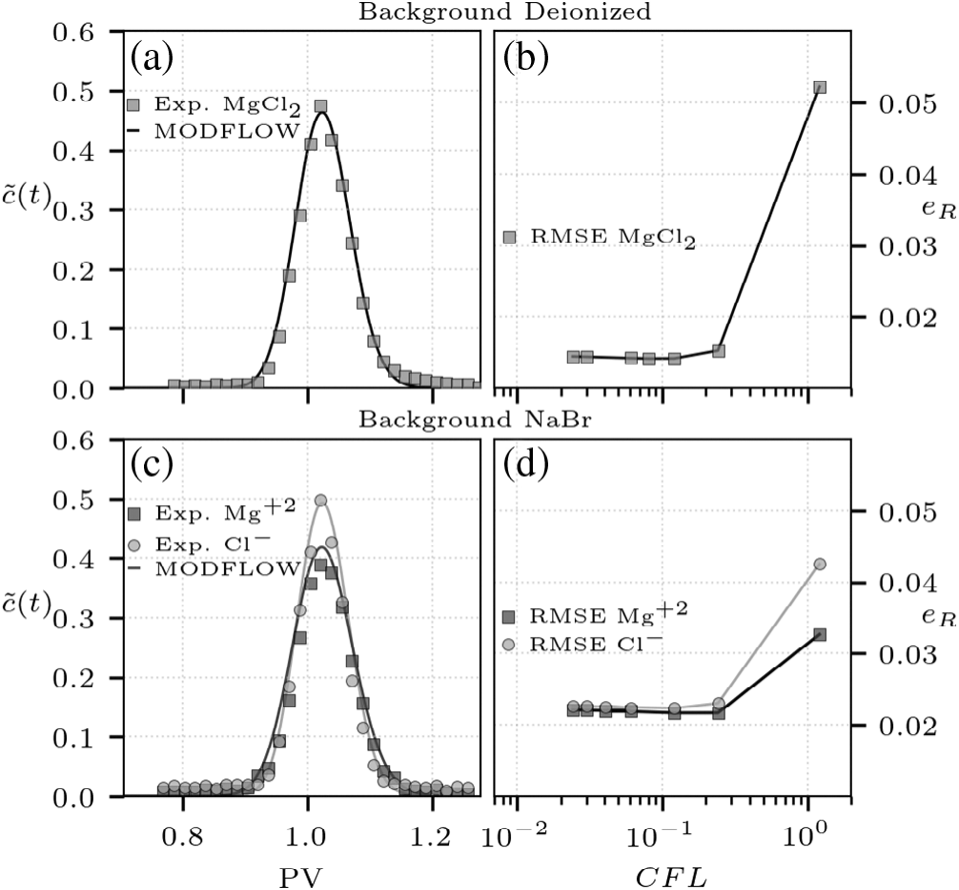
Comparison of flux‐normalized breakthrough concentrations $\tilde{c}\left(t\right)$ at the outlet of the homogeneous flow‐through system for the pulse‐injection tracer test. Panel (a) shows the ion breakthrough for the case with deionized water, and panel (b) the corresponding RMSE with respect to the experimental data for simulations with different time steps. Panel (c) shows the ion breakthrough for the injection into the medium saturated with NaBr solution, and panel (d) the corresponding RMSE with respect to the experimental data for each ion, for different time steps. In panels (a, c), symbols represent the data collected from multispecies ionic transport laboratory experiments (Muniruzzaman and Rolle 2017), and the lines are the results of the proposed MODFLOW‐API approach. Simulations consider a uniform flow velocity v=4.83 m/d.

An additional analysis performed in the homogeneous setup is the discussion of transient transport and solute breakthrough curves at the outlet of the domain. To this end, simulations of a pulse injection are benchmarked with breakthrough data obtained from multispecies ionic transport experiments (shown in Muniruzzaman and Rolle 2017). As before, the flow‐through system is either saturated with deionized water or with a NaBr solution acting as background electrolyte. In this case, tracer injection is performed from a line source of width $w_{\text{in}}=2$ cm, releasing the inflow concentrations for a prescribed duration of $t_{\text{in}}=15$ min effectively representing a pulse injection, and MgCl_2_ is considered as tracer solution. Breakthrough concentrations for the different ions are calculated at the end of the experimental setup, normalized by the solute‐injection flux (Muniruzzaman and Rolle 2017). A comparison between results from this work and the reference experimental data is shown in Figure 4. For the condition of injection into deionized environment the MODFLOW‐API model is capable of representing the coupled breakthrough curve, with both ion concentrations following the same trend in time due to the coupling of their dispersive fluxes both in the longitudinal and transverse directions (Figure 4a). Likewise, simulations for the case with a background NaBr electrolyte now show differences between the breakthrough curves, consequence of the differences in self‐dispersion coefficients of the tracer ions (Figure 4c). These transient transport simulations also serve the purpose of evaluating the performance of the proposed API workflow based on a *lagged‐one‐time‐step* approach. To this end, numerical simulations were performed considering different magnitudes of the time step Δt, and the simulated breakthrough is compared to the experimental data by means of the root mean squared error (RMSE, $e_{R}$). In this case, simulations are characterized by the Courant–Friedrichs–Lewy number CFL=vΔt/Δx, with values in between CFL∈[0.01,1]. For both experimental conditions (Figure 4b and 4d), the RMSE shows that the numerical simulation is in its best agreement with the experimental data for the time steps associated to CFL≤0.1. This is a typical reference value to be used in advection‐dominated transient transport simulations, and the error metric remains stable when further decreasing the time step from this threshold. The results presented in this section are overall in good agreement with the reference data, demonstrating the successful incorporation of electrostatic coupling into a MODFLOW‐based multispecies solute transport simulation, both for steady‐state and transient transport conditions.

### Transport through Heterogeneous Media

Numerical simulations were also performed considering heterogeneities in the porous medium. In this section, the objective is to validate the MODFLOW implementation via comparison with multispecies ionic transport simulations in a streamline‐oriented framework previously discussed in the literature, validated with experimental data (Muniruzzaman et al. 2014). In these models, a flow‐through system with inclusions of different permeability (Figure 2b and 2c) is employed to study multispecies ionic transport through heterogeneous domains. There are two different configurations of heterogeneity: (i) the porous matrix is modified with an inclusion of lower permeability (labeled as HET A; Figure 2b), and (ii) a different setup with two higher permeability inclusions (labeled as HET B; Figure 2c). In both cases, the flow model is at steady‐state and transport simulations with MODFLOW were verified to be performed with groundwater flow velocities consistent with those obtained from the streamline‐oriented code (as shown in *Velocities in heterogeneous flow‐through domains* in Appendix S1). Transport simulations with MODFLOW are advanced in time using the API, following the same coupling approach previously discussed. The initial assessment for these experimental conditions is made based on the simulation of NaCl as a tracer in the deionized environment. As before, Na^+^ and Cl^−^ are expected to displace together in order to maintain charge balance and this behavior was verified to occur in results from both the streamline‐oriented code and MODFLOW, effectively preserving neutral charge balance. The latter is easily verified by evaluating the electroneutrality condition (Equation 1) with the ion concentrations at a given instant.

**Figure 5:**
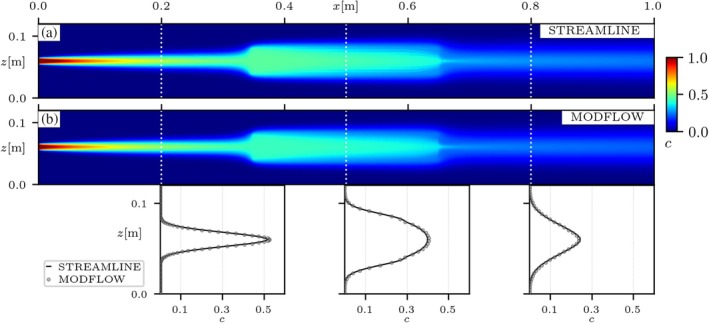
Comparison of Na^+^ concentration maps for the injection of NaCl into the porous medium with a lower‐permeability inclusion (HET A in Figure 2), initially saturated with pure water. (a) Concentrations from the streamline‐oriented code (Muniruzzaman et al. 2014), and (b) concentrations from the proposed MODFLOW‐API approach. Transverse concentration profiles are shown for coordinates x={0.2,0.5,0.8}[m]. Concentrations of Cl^−^ follow the same distribution than of Na^+^ due to their electrostatic coupling.

A comparison of the concentrations obtained from the streamline‐oriented code with those from MODFLOW is shown in Figure 5, simulating transport through the heterogeneous medium with a lower‐permeability inclusion (HET A). Transverse concentration profiles at different cross‐sections of the flow‐through system are in good agreement between the two simulation approaches. The same simulations were also performed in the scenario with higher‐permeability inclusions (HET B) and it was verified that the MODFLOW‐based results were also in agreement with the reference streamline‐oriented simulations (refer to *Simulated concentrations in HET B* in Appendix S1). These results support the validity of the MODFLOW‐based implementation and its applicability to flow‐through systems with relatively simple hydraulic heterogeneity. A matter of discussion in these simulations was the fact that in heterogeneous systems streamlines can easily deviate from the principal directions of the grid, a consequence of hydraulic heterogeneities, challenging the performance of the *Simplified Formulation* of dispersion. However, the heterogeneous simulations in this section suggest that errors have a negligible influence, as evidenced by the consistent agreement between MODFLOW and the reference streamline‐oriented simulations.

**Figure 6:**
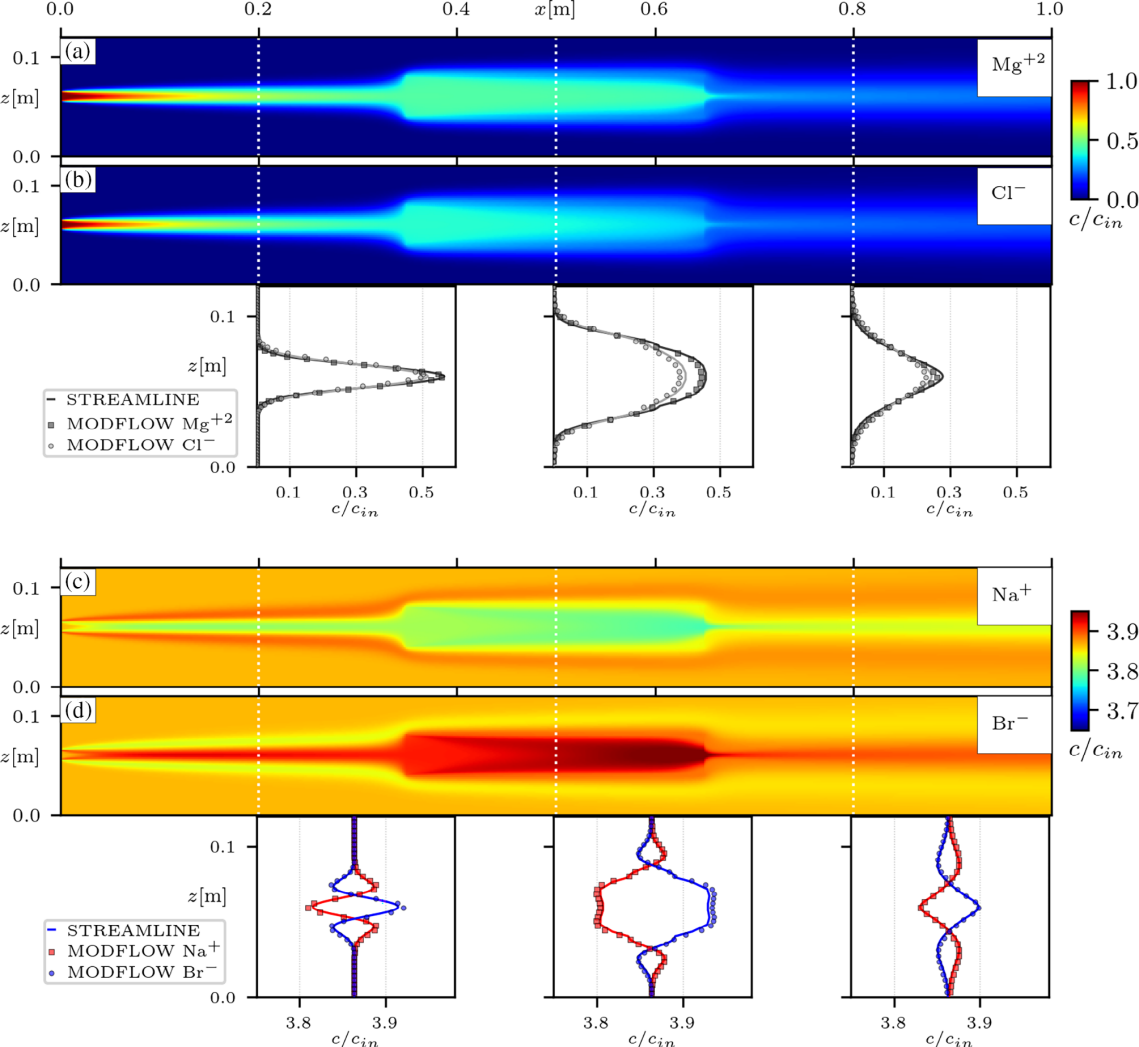
Results from simulation of tracer MgCl_2_ into NaBr background solution in the heterogeneous medium with a lower‐permeability inclusion (HET A). Panels (a, b) show the distribution of concentrations for the tracer salt ions (${\mathrm{Mg}}^{2+},{\mathrm{Cl}}^{-}$), with their corresponding transverse concentration profiles at cross‐sections x∈{0.2,0.5,0.8}[m]. Panels (c, d) present the same for the ions of the background solution (${\mathrm{Na}}^{+},{\mathrm{Br}}^{-}$). $c_{\text{in}}$ stands for the concentration of the injected tracer. The concentration maps were computed with the proposed MODFLOW‐API approach. In the transverse concentration profiles, solid lines show the reference simulations with the streamline‐oriented code (Muniruzzaman et al. 2014) and the symbols are the results from the proposed MODFLOW‐API approach.

**Figure 7:**
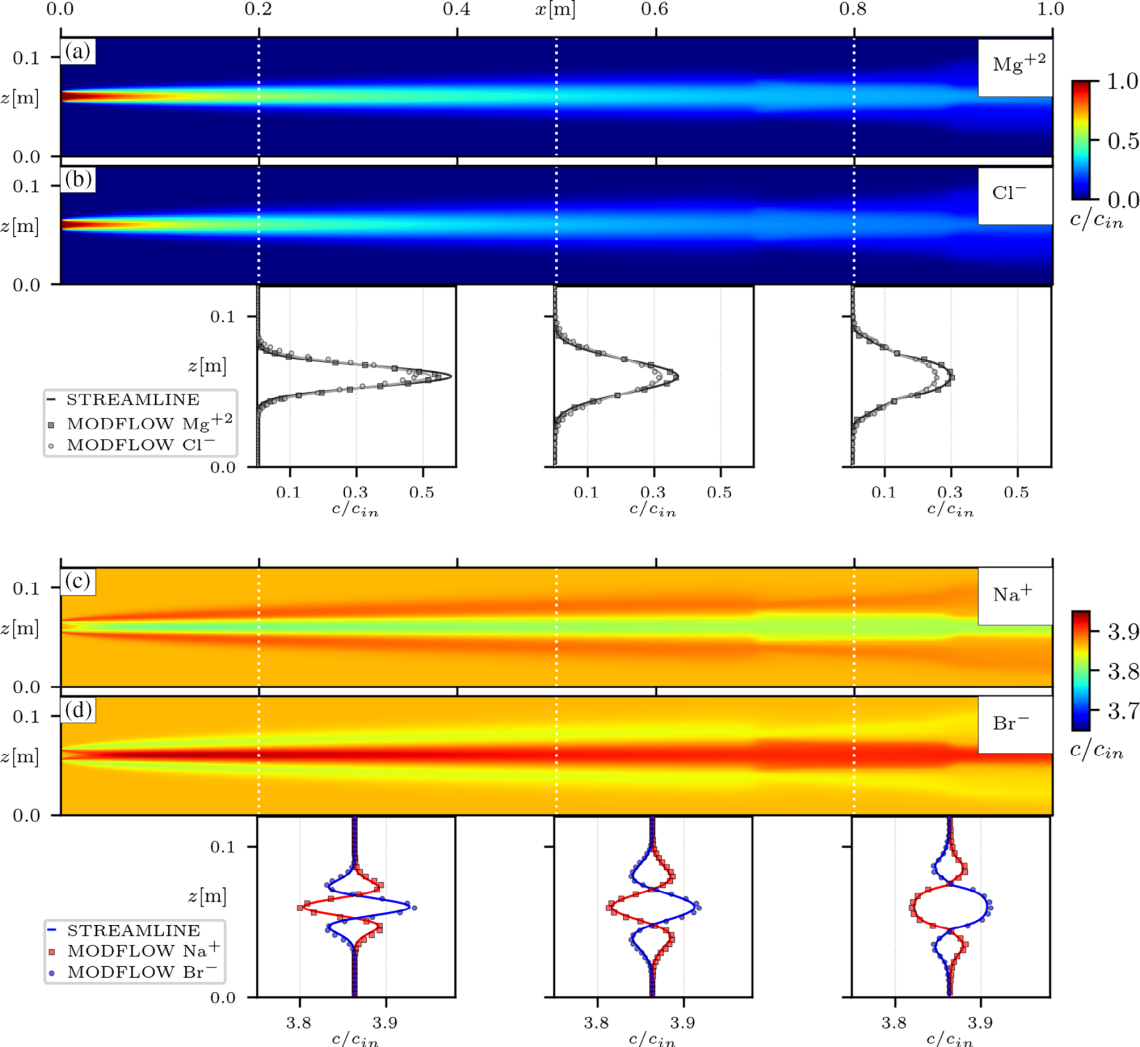
Results from simulation of tracer MgCl_2_ into NaBr background solution in the heterogeneous medium with two higher‐permeability inclusions (HET B). Panels (a, b) show the distribution of concentrations for the tracer salt ions (${\mathrm{Mg}}^{+2},{\mathrm{Cl}}^{-}$), with their corresponding transverse concentration profiles at cross‐sections x∈{0.2,0.5,0.8}[m]. Panels (c, d) present the same for the ions of the background solution (${\mathrm{Na}}^{+},{\mathrm{Br}}^{-}$). $c_{\text{in}}$ stands for the concentration of the injected tracer. The concentration maps were computed with the proposed MODFLOW‐API approach. In the transverse concentration profiles, solid lines show the reference simulations with the streamline‐oriented code (Muniruzzaman et al. 2014) and the symbols are the results from the proposed MODFLOW‐API approach.

Transport simulations with a strong background electrolyte solution were also performed in the two heterogeneous setups, emulating the injection of the tracer salt ${\text{MgCl}}_{2}$. The system is initially saturated with a solution of NaBr at a concentration of about four times higher than the tracer. Figure 6 presents the steady‐state concentrations for all ions in solution coupled with the MODFLOW‐API, in the porous medium with a lower‐permeability inclusion (HET A). In this configuration, the electrolyte ions can disperse closer to their liberated state as the buffer electrolyte concentration can also compensate to maintain the pore water electroneutrality. In this case, differences in ion concentrations are now visible due to differences in dispersion coefficients, as seen in the transverse concentration profiles (Figure 6a and 6b). This behavior is consistently achieved with the MODFLOW model, with concentrations of both the tracer and background ions in agreement with the reference streamline‐oriented model. The same is confirmed for the heterogeneous medium with higher‐permeability inclusions (HET B), as seen in Figure 7. This exercise further confirms the validity of the MODFLOW‐based simulations, with numerical results consistently in agreement with the reference model, for both conditions of heterogeneity, and while accounting for different scenarios of background electrolyte solution. Furthermore, these results showcase a new simulation capability in the context of the MODFLOW framework, achieved by extending the functionalities of the native solute transport solver via the MODFLOW‐API. Results show that the coupling strategy discussed in this work is effective in incorporating electrostatic coupling effects into multispecies solute transport simulations with MODFLOW.

## Conclusions

Electrostatic interactions occur during the transport of ionic chemical species, and their incorporation into solute transport codes has received increasing attention in recent years, aiming to improve the accuracy of simulators in applications where electrostatic coupling effects are known to be of relevance. This physical process is rooted in the different species‐specific diffusion coefficients, the different electric charge of ionic species, and in the electroneutrality of pore water solutions. This work discussed the incorporation of electrostatic coupling effects into multispecies solute transport simulations performed with the transport module of MODFLOW. Although the program is a well‐known simulator in hydrogeology, the incorporation of electrostatic effects remained unexplored. Making use of the native packages of MODFLOW, and the Application Programming Interface (MODFLOW‐API; Hughes et al. 2022) it was possible to implement a simulation workflow, based on a *lagged‐one‐time‐step* approach, effectively incorporating electrostatic interactions into multispecies solute transport simulations with the program. In this context, MODFLOW‐based simulations of multispecies ionic solute transport were in agreement with available benchmark solutions, supporting the validity of the coupling strategy. Results showed that the model was effective in reproducing the reference ion concentrations when benchmarked in problems of transport through a porous medium with simple hydraulic heterogeneity. Along this line, one potential limitation of the implementation discussed in this work is the use of the *Simplified Formulation* of dispersion, which has some known limitations while simulating solute transport in cases where the flow is highly heterogeneous. Still, the simplified approach provided a reasonable and straightforward path to evaluate the feasibility of incorporating electrostatic coupling effects into the program, and future developments could be dedicated to integrating the coupling into the more generalized XT3D formulation of dispersion (Provost et al. 2017; Langevin et al. 2022), which was not discussed in this paper. This work is innovative since the integration of electrostatic coupling into multispecies solute transport simulations with MODFLOW opens new perspectives of potential applications with the program. In particular, the implementation of the electrostatic coupling effect is flexible because calculations make use of the internal cell‐connectivity arrays of MODFLOW. This means that multispecies ionic transport simulations could be performed in hydrological systems already simulated with the program, that make use of the different stress packages to represent natural or engineered aquifer conditions (e.g., injection/abstraction wells, recharge, river‐groundwater interactions, and others). Likewise, the presented MODFLOW‐API workflow could be extended to accommodate charge interactions not only in pore water, but also at surface‐solution interfaces aiming to describe transport in chemically and electrostatically heterogeneous porous media (e.g., sandy–clayey systems). Furthermore, one could consider also extending the API workflow to achieve the coupling with geochemical simulators in order to extend the capabilities of the program to simulate reactive multispecies ionic transport.

## Authors' Note

The authors do not have any conflicts of interest or financial disclosures to report.

## Supporting information


**Appendix S1.** Additional supporting figures validating flow‐velocities and concentrations in heterogeneous flow‐through simulations.

## Data Availability

Codes used for simulations, implementing the workflow for electrostatic coupling of ionic species, are freely available at https://github.com/tuda‐aqgeochemistry/modflow‐electrostatic‐coupling.

## References

[gwat70033-bib-0001] Appelo, C. , A. Vinsot , S. Mettler , and S. Wechner . 2008. Obtaining the porewater composition of a clay rock by modeling the in‐ and out‐diffusion of anions and cations from an in‐situ experiment. Journal of Contaminant Hydrology 101, no. 1–4: 67–76. 18805602 10.1016/j.jconhyd.2008.07.009

[gwat70033-bib-0002] Appelo, C.A.J. , and P. Wersin . 2007. Multicomponent diffusion modeling in clay systems with application to the diffusion of tritium, iodide, and sodium in opalinus clay. Environmental Science & Technology 41, no. 14: 5002–5007. 17711215 10.1021/es0629256

[gwat70033-bib-0003] Atteia, O. , H. Prommer , D. Vlassopoulos , L. André , and G. Cohen . 2023. muFlowReacT: A library to solve multiphase multicomponent reactive transport on unstructured meshes. Groundwater 62, no. 3: 357–370. 10.1111/gwat.13345 37522260

[gwat70033-bib-0004] Basilio Hazas, M. , F. Ziliotto , J. Lee , M. Rolle , and G. Chiogna . 2023. Evolution of plume geometry, dilution and reactive mixing in porous media under highly transient flow fields at the surface water‐groundwater interface. Journal of Contaminant Hydrology 258: 104243. 37696230 10.1016/j.jconhyd.2023.104243

[gwat70033-bib-0005] Bear, J. 1979. Hydraulics of Groundwater. McGraw‐Hill Series in Water Resources and Environmental Engineering. New York: McGraw‐Hill.

[gwat70033-bib-0006] Boudreau, B.P. , F.J. Meysman , and J.J. Middelburg . 2004. Multicomponent ionic diffusion in porewaters: Coulombic effects revisited. Earth and Planetary Science Letters 222, no. 2: 653–666.

[gwat70033-bib-0007] Carey, A.E. , S.W. Wheatcraft , R.J. Glass , and J.P. O'Rourke . 1995. Non‐Fickian ionic diffusion across high‐concentration gradients. Water Resources Research 31, no. 9: 2213–2218.

[gwat70033-bib-0008] Charlet, L. , P. Alt‐Epping , P. Wersin , and B. Gilbert . 2017. Diffusive transport and reaction in clay rocks: A storage (nuclear waste, CO_2_, H_2_), energy (shale gas) and water quality issue. Advances in Water Resources 106: 39–59.

[gwat70033-bib-0009] Chiogna, G. , C. Eberhardt , P. Grathwohl , O.A. Cirpka , and M. Rolle . 2010. Evidence of compound‐dependent hydrodynamic and mechanical transverse dispersion by multitracer laboratory experiments. Environmental Science & Technology 44, no. 2: 688–693. 20020677 10.1021/es9023964

[gwat70033-bib-0010] Cussler, E.L. 2009. Diffusion: Mass Transfer in Fluid Systems. New York: Cambridge University Press.

[gwat70033-bib-0011] Giambalvo, E.R. , C.I. Steefel , A.T. Fisher , N.D. Rosenberg , and C. Wheat . 2002. Effect of fluid‐sediment reaction on hydrothermal fluxes of major elements, eastern flank of the Juan de Fuca Ridge. Geochimica et Cosmochimica Acta 66, no. 10: 1739–1757.

[gwat70033-bib-0012] Guedes de Carvalho, J. , and J. Delgado . 2005. Overall map and correlation of dispersion data for flow through granular packed beds. Chemical Engineering Science 60, no. 2: 365–375.

[gwat70033-bib-0013] Hochstetler, D.L. , M. Rolle , G. Chiogna , C.M. Haberer , P. Grathwohl , and P.K. Kitanidis . 2013. Effects of compound‐specific transverse mixing on steady‐state reactive plumes: Insights from pore‐scale simulations and Darcy‐scale experiments. Advances in Water Resources 54: 1–10.

[gwat70033-bib-0014] Huang, P.‐W. , B. Flemisch , C.‐Z. Qin , M.O. Saar , and A. Ebigbo . 2023. Validating the Nernst–Planck transport model under reaction‐driven flow conditions using RetroPy v1.0. Geoscientific Model Development 16, no. 16: 4767–4791.

[gwat70033-bib-0015] Hughes, J.D. , M.J. Russcher , C.D. Langevin , E.D. Morway , and R.R. McDonald . 2022. The MODFLOW application programming Interface for simulation control and software interoperability. Environmental Modelling & Software 148: 105257.

[gwat70033-bib-0016] Jin, B. , M. Rolle , T. Li , and S.B. Haderlein . 2014. Diffusive fractionation of BTEX and chlorinated ethenes in aqueous solution: Quantification of spatial isotope gradients. Environmental Science & Technology 48, no. 11: 6141–6150. 24811111 10.1021/es4046956

[gwat70033-bib-0017] Kurotori, T. , C. Zahasky , S.A. Hosseinzadeh Hejazi , S.M. Shah , S.M. Benson , and R. Pini . 2019. Measuring, imaging and modelling solute transport in a microporous limestone. Chemical Engineering Science 196: 366–383.

[gwat70033-bib-0018] Langevin, C.D. , J.D. Hughes , A.M. Provost , M.J. Russcher , and S. Panday . 2023. MODFLOW as a Configurable Multi‐Model Hydrologic Simulator. Groundwater 62, no. 1: 111–123. 10.1111/gwat.13351 37656806

[gwat70033-bib-0019] Langevin, C.D. , A.M. Provost , S. Panday , and J.D. Hughes . 2022. Documentation for the MODFLOW 6 Groundwater Transport Model. U.S. Geological Survey Techniques and Methods 6‐A61. U.S. Geological Survey: Reston, VA. 10.3133/tm6a61

[gwat70033-bib-0020] Langevin, C.D. , J.D. Hughes , E.R. Banta , R.G. Niswonger , S. Panday , and A.M. Provost . 2017. Documentation for the MODFLOW 6 Groundwater Flow Model, U.S. Geological Survey Techniques and Methods 6‐A55. U.S. Geological Survey: Reston, VA. 10.3133/tm6A55

[gwat70033-bib-0021] Larsen, J.D. , C.D. Langevin , J.D. Hughes , and R.G. Niswonger . 2024. An agricultural package for MODFLOW 6 using the application programming interface. Groundwater 62, no. 1: 157–166. 10.1111/gwat.13367 37882370

[gwat70033-bib-0022] Liu, C. , J. Shang , and J.M. Zachara . 2011. Multispecies diffusion models: A study of uranyl species diffusion. Water Resources Research 47, no. 12: W12514.

[gwat70033-bib-0023] López‐Vizcaíno, R. , V. Cabrera , R. Sprocati , M. Muniruzzaman , M. Rolle , V. Navarro , and Á. Yustres . 2022. A modeling approach for electrokinetic transport in double‐porosity media. Electrochimica Acta 431: 141139.

[gwat70033-bib-0024] Muniruzzaman, M. , and M. Rolle . 2021. Impact of diffuse layer processes on contaminant forward and back diffusion in heterogeneous sandy‐clayey domains. Journal of Contaminant Hydrology 237: 103754. 33517148 10.1016/j.jconhyd.2020.103754

[gwat70033-bib-0025] Muniruzzaman, M. , and M. Rolle . 2019. Multicomponent ionic transport modeling in physically and electrostatically heterogeneous porous media with PhreeqcRM coupling for geochemical reactions. Water Resources Research 55, no. 12: 11121–11143.

[gwat70033-bib-0026] Muniruzzaman, M. , and M. Rolle . 2017. Experimental investigation of the impact of compound‐specific dispersion and electrostatic interactions on transient transport and solute breakthrough. Water Resources Research 53, no. 2: 1189–1209.

[gwat70033-bib-0027] Muniruzzaman, M. , C.M. Haberer , P. Grathwohl , and M. Rolle . 2014. Multicomponent ionic dispersion during transport of electrolytes in heterogeneous porous media: Experiments and model‐based interpretation. Geochimica et Cosmochimica Acta 141: 656–669.

[gwat70033-bib-0028] Özişik, M. , H. Orlande , M. Colaço , and R. Cotta . 2017. Finite Difference Methods in Heat Transfer, Second ed. CRC Press: Boca Raton, FL.

[gwat70033-bib-0029] Parkhurst, D.L. , and L. Wissmeier . 2015. PhreeqcRM: A reaction module for transport simulators based on the geochemical model PHREEQC. Advances in Water Resources 83: 176–189.

[gwat70033-bib-0030] Parkhurst, D.L. , and C. Appelo . 2013. Description of Input and Examples for PHREEQC Version 3: A Computer Program for Speciation, Batch‐Reaction, One‐Dimensional Transport, and Inverse Geochemical Calculations. U.S. Geological Survey Techniques and Methods 6‐A43. U.S. Geological Survey: Reston, VA. 10.3133/tm6A43

[gwat70033-bib-0031] Pérez‐Illanes, R. , and D. Fernàndez‐Garcia . 2024. MODPATH‐RW: A random walk particle tracking code for solute transport in heterogeneous aquifers. Groundwater 62: 617–634. 10.1111/gwat.13390 38279644

[gwat70033-bib-0032] Pérez‐Illanes, R. , M.W. Saaltink , and D. Fernàndez‐Garcia . 2024. Nonlinear formulation of multicomponent reactive transport with species‐specific dispersion properties. Water Resources Research 60, no. 3: e2023WR036358.

[gwat70033-bib-0033] Provost, A.M. , C.D. Langevin , and J.D. Hughes . 2017. Documentation for the “XT3D” Option in the Node Property Flow (NPF) Package of MODFLOW 6. U.S. Geological Survey Techniques and Methods 6‐A56. U.S. Geological Survey: Reston, VA. 10.3133/tm6A56

[gwat70033-bib-0034] Rasouli, P. , C.I. Steefel , K.U. Mayer , and M. Rolle . 2015. Benchmarks for multicomponent diffusion and electrochemical migration. Computational Geosciences 19, no. 3: 523–533.

[gwat70033-bib-0035] Rolle, M. , R. Sprocati , M. Masi , B. Jin , and M. Muniruzzaman . 2018. Nernst‐Planck‐based description of transport, coulombic interactions, and geochemical reactions in porous media: Modeling approach and benchmark experiments. Water Resources Research 54, no. 4: 3176–3195.

[gwat70033-bib-0036] Rolle, M. , and P.K. Kitanidis . 2014. Effects of compound‐specific dilution on transient transport and solute breakthrough: A pore‐scale analysis. Advances in Water Resources 71: 186–199.

[gwat70033-bib-0037] Rolle, M. , M. Muniruzzaman , C.M. Haberer , and P. Grathwohl . 2013. Coulombic effects in advection‐dominated transport of electrolytes in porous media: Multicomponent ionic dispersion. Geochimica et Cosmochimica Acta 120: 195–205.

[gwat70033-bib-0038] Rolle, M. , D. Hochstetler , G. Chiogna , P.K. Kitanidis , and P. Grathwohl . 2012. Experimental investigation and pore‐scale modeling interpretation of compound‐specific transverse dispersion in porous media. Transport in Porous Media 93, no. 3: 347–362.

[gwat70033-bib-0039] Soler, J.M. , C.I. Steefel , T. Gimmi , O.X. Leupin , and V. Cloet . 2019. Modeling the ionic strength effect on diffusion in clay. The DR‐A experiment at Mont Terri. ACS Earth and Space Chemistry 3, no. 3: 442–451.

[gwat70033-bib-0040] Sprocati, R. , A. Gallo , R. Sethi , and M. Rolle . 2020. Electrokinetic delivery of reactants: Pore water chemistry controls transport, mixing, and degradation. Environmental Science & Technology 55, no. 1: 719–729. 33295762 10.1021/acs.est.0c06054

[gwat70033-bib-0041] Sprocati, R. , M. Masi , M. Muniruzzaman , and M. Rolle . 2019. Modeling electrokinetic transport and biogeochemical reactions in porous media: A multidimensional Nernst–Planck–Poisson approach with PHREEQC coupling. Advances in Water Resources 127: 134–147.

[gwat70033-bib-0042] Steefel, C.I. , and C. Tournassat . 2020. A model for discrete fracture‐clay rock interaction incorporating electrostatic effects on transport. Computational Geosciences 25, no. 1: 395–410.

[gwat70033-bib-0043] Steefel, C.I. , C.A.J. Appelo , B. Arora , D. Jacques , T. Kalbacher , O. Kolditz , V. Lagneau , P. Lichtner , K.U. Mayer , J.C.L. Meeussen , S. Molins , J.D. Moulton , H. Shao , J.S. Jiri , N. Spycher , S.B. Yabusaki , and G.‐T. Yeh . 2014. Reactive transport codes for subsurface environmental simulation. Computational Geosciences 19, no. 3: 445–478.

[gwat70033-bib-0044] Trinchero, P. , A. Nardi , O. Silva , and P. Bruch . 2022. Simulating electrochemical migration and anion exclusion in porous and fractured media using PFLOTRANNP. Computers & Geosciences 166: 105166.

[gwat70033-bib-0045] Tournassat, C. , C.I. Steefel , and T. Gimmi . 2020. Solving the Nernst‐Planck equation in heterogeneous porous media with finite volume methods: Averaging approaches at interfaces. Water Resources Research 56, no. 3: e2019WR026832.

[gwat70033-bib-0046] Tournassat, C. , and C.I. Steefel . 2019. Modeling diffusion processes in the presence of a diffuse layer at charged mineral surfaces: A benchmark exercise. Computational Geosciences 25, no. 4: 1319–1336.

[gwat70033-bib-0047] Urík, J. , A. Paschke , and B. Vrana . 2020. Diffusion coefficients of polar organic compounds in agarose hydrogel and water and their use for estimating uptake in passive samplers. Chemosphere 249: 126183. 32088466 10.1016/j.chemosphere.2020.126183

[gwat70033-bib-0048] White, J.T. , J.E. Ewing , G. Ruskauff , and H. Rashid . 2023. A generic closed‐loop contaminant treatment system package for MODFLOW 6. Groundwater 61, no. 1: 131–138. 10.1111/gwat.13239 36594877

[gwat70033-bib-0049] Wienkenjohann, H. , B. Jin , and M. Rolle . 2023. Diffusive‐dispersive isotope fractionation of chlorinated ethenes in groundwater: The key role of incomplete mixing and its multi‐scale effects. Water Resources Research 59, no. 4: e2022WR034041.

[gwat70033-bib-0050] Wilke, C.R. , and P. Chang . 1955. Correlation of diffusion coefficients in dilute solutions. AICHE Journal 1, no. 2: 264–270.

[gwat70033-bib-0051] Worch, E. 1993. A new equation for the calculation of diffusion coefficients for dissolved substances. Vom Wasser 81: 289–297.

[gwat70033-bib-0052] Wu, M.Z. , V.E. Post , S.U. Salmon , E.D. Morway , and H. Prommer . 2015. PHT3D‐UZF: A reactive transport model for variably‐saturated porous media. Groundwater 54, no. 1: 23–34. 10.1111/gwat.12318 25628017

[gwat70033-bib-0053] Ye, Y. , S. Liu , G. Chiogna , C. Lu , and M. Rolle . 2025. Density effects on mixing in porous media: Multi‐dimensional flow‐through experiments and model‐based interpretation. Transport in Porous Media 152, no. 4: 24.

[gwat70033-bib-0054] Ye, Y. , G. Chiogna , O. Cirpka , P. Grathwohl , and M. Rolle . 2015. Experimental investigation of compound‐specific dilution of solute plumes in saturated porous media: 2‐D vs. 3‐D flow‐through systems. Journal of Contaminant Hydrology 172: 33–47. 25462641 10.1016/j.jconhyd.2014.11.002

