# Supplementary material for: Incorporating Electrostatic Coupling Effects into Multispecies Solute Transport Simulations with MODFLOW
Source: Ground Water. 2025 Nov 28;64(2):210–22. doi: 10.1111/gwat.70033 (PMC12990972; doi:10.1111/gwat.70033)
Supplement: Supplementary file 1 — Appendix S1. Additional supporting figures validating flow‐velocities and concentrations in heterogeneous flow‐through simulations. [file GWAT-64-210-s001.pdf]

Methods Brief/

# Supporting Information: Incorporating Electrostatic Coupling Effects into Multispecies Solute Transport Simulations with MODFLOW

Rodrigo Pérez-Illanes

Corresponding author: Aquatic Geochemistry Group, Institute of Applied Geosciences, Technical University of Darmstadt, Schnittspahnstraße 9, 64287 Darmstadt, Germany.

rodrigo.perez@tu-darmstadt.de

Christian D. Langevin

S.S. Papadopulos & Assoc., Inc., 12 Dove Ln, Saint Paul, MN, 55127, United States.

langevin@sspa.com

Muhammad Muniruzzaman

Institute of Geosciences, University of Bonn, Kirschallee 1-3, 53115 Bonn, Germany.

Geological Survey of Finland, Vuorimiehentie 5, 02151 Espoo, Finland.

m.muniruzzaman@uni-bonn.de

Massimo Rolle

Aquatic Geochemistry Group, Institute of Applied Geosciences, Technical University of Darmstadt, Schnittspahnstraße 9, 64287 Darmstadt, Germany.

massimo.rolle@tu-darmstadt.de

# Abstract

This article elaborates on the topic of electrostatic coupling of charged species in saturated porous media and presents a methodology for incorporating the effect into multispecies solute transport simulations with MODFLOW. The integration is achieved through the Application Programming Interface of the program (MODFLOW-API) which enables the access to concentrations and dispersion coefficients of all species during the simulation. These are necessary to calculate a dispersive correction that effectively incorporates electrostatic coupling into the model. Numerical results demonstrate the effectiveness of the coupling strategy, benchmarking the implementation with previously validated numerical simulators and with experimental data. In the following, details on the velocity calculations in heterogeneous domains, and additional results for the multispecies ionic transport simulations are provided.


# Velocities in heterogeneous flow-through domains

Comparison of spatial distribution of velocities obtained with the streamline-oriented code (Muniruzzaman et al. 2014) and the proposed MODFLOW-API approach. Figure (S.1) is the distribution for the medium HET A, and Figure (S.2) for the porous medium HET B. It is verified that both simulation approaches lead to a consistent distribution of flow velocities, which is also confirmed via the agreement in transverse velocity profiles.

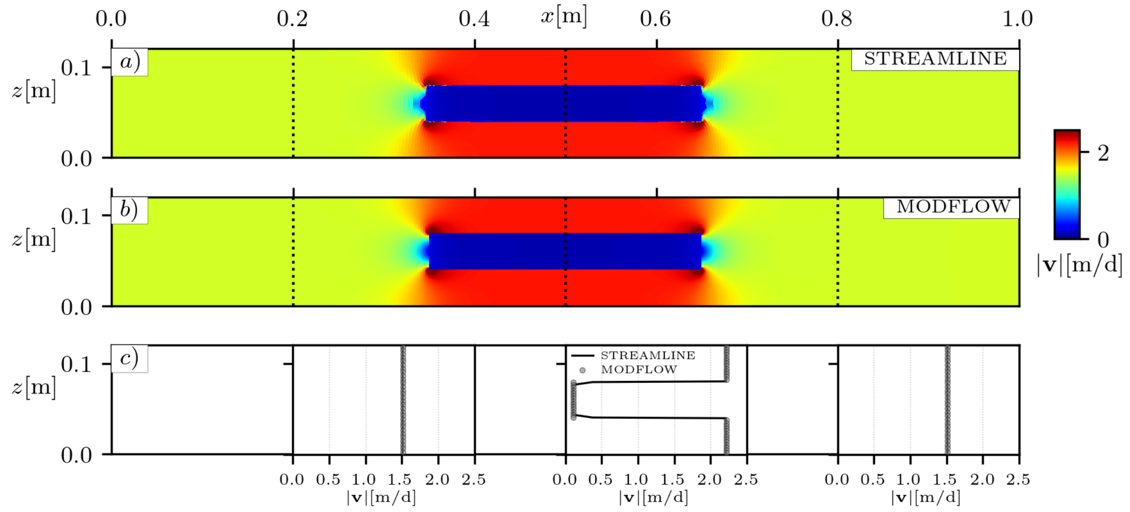


Figure S.1: Comparison of velocities in setup HET A obtained from *a*) the streamline-oriented code, and *b*) MODFLOW. *c*) Transverse profiles of velocity at $x = \{0.2, 0.5, 0.8\}$[m]

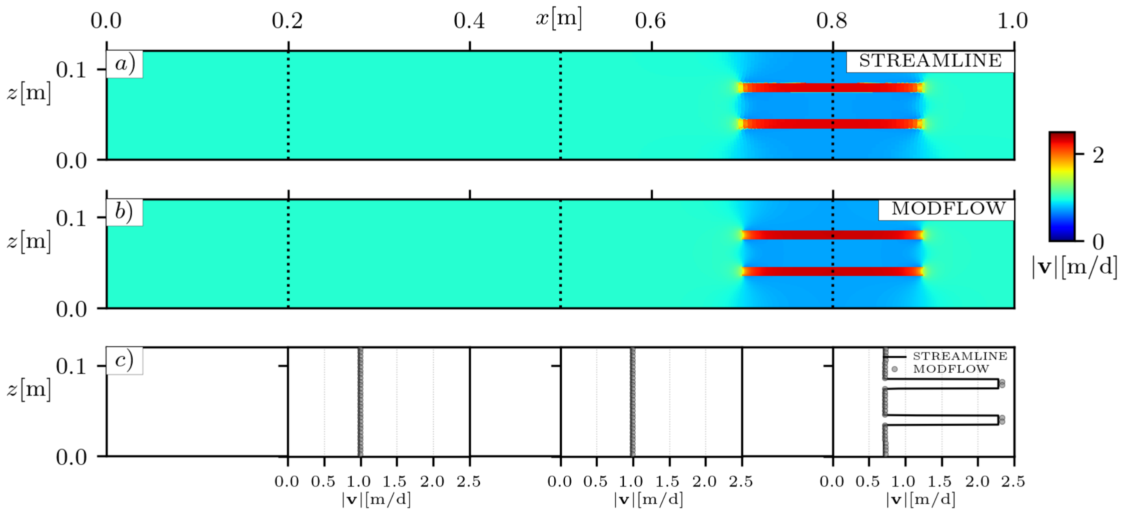


Figure S.2: Comparison of velocities in setup HET B obtained from *a*) the streamline-oriented code, and *b*) MODFLOW. *c*) Transverse profiles of velocity at $x = \{0.2, 0.5, 0.8\}$[m]

# Simulated concentrations in HET B

Comparison of concentrations obtained from the streamline-oriented code (Muniruzzaman et al. 2014) with those generated with the proposed MODFLOW-API approach, while simulating NaCl injection into the heterogeneous setup with higher-permeability inclusions (HET B).

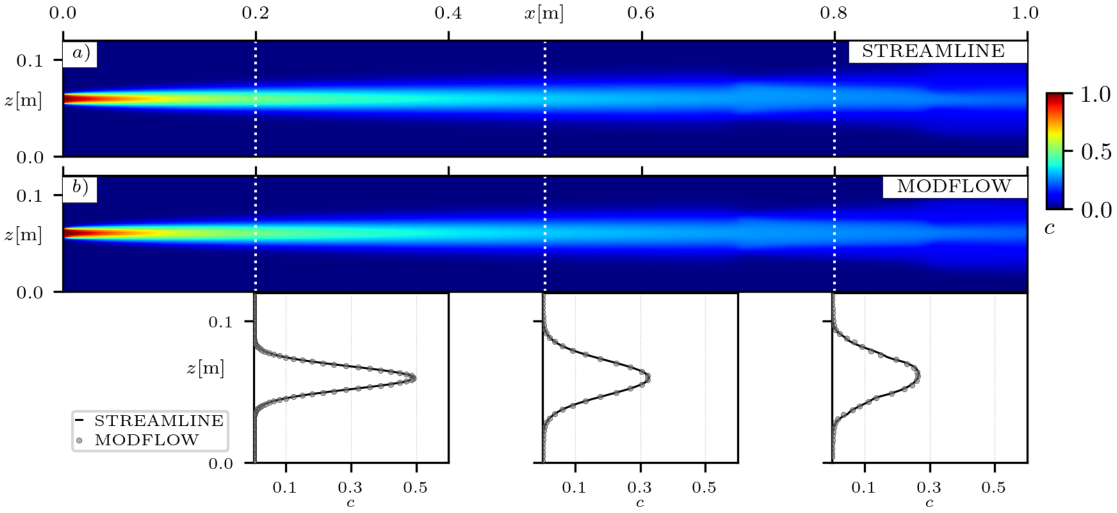


Figure S.3: Comparison of $Na^+$ concentration maps for the injection of NaCl into the porous medium with two higher-permeability inclusions (HET B), initially saturated with pure water. *a*) Concentrations from the streamline-oriented code, and *b*) concentrations from MODFLOW. Transverse concentration profiles are shown for coordinates $x = \{0.2, 0.5, 0.8\}$[m]. Concentrations of $Cl^-$ follow the same distribution than of $Na^+$ due to their electrostatic coupling.

# References

Muniruzzaman, M., C. M. Haberer, P. Grathwohl, and M. Rolle. 2014. Multicomponent ionic dispersion during transport of electrolytes in heterogeneous porous media: Experiments and model-based interpretation. *Geochimica et Cosmochimica Acta* 141, 656–669.
